# Comparison between virtual and hybrid education for psychological factors and academic stress in freshman nursing students: a case-control study

**DOI:** 10.1186/s12912-023-01477-5

**Published:** 2023-09-04

**Authors:** Marta Elena Losa-Iglesias, César Calvo-Lobo, Raquel Jiménez-Fernández, David Rodríguez-Sanz, Inmaculada Corral-Liria, Israel Casado-Hernández, Ricardo Becerro-de-Bengoa-Vallejo

**Affiliations:** 1 Faculty of Health Sciences, Universidad Rey Juan Carlos, Alcorcon, 28922 Spain; 2 Facultad de Enfermería, Fisioterapia y Podología, Universidad Complutense de Madrid, Plaza Ramón y Cajal, 3, Madrid, 28040 Spain

**Keywords:** Learning, Nursing students, Pandemics, Stress, psychological.

## Abstract

**Background:**

The coronavirus 2019 (COVID-19) pandemic has prompted several changes in the learning methods of students. The purpose of this study was to establish whether a relationship between levels of resilience, self-esteem, anxiety, depression, and academic stress in both hybrid and virtual learning education exist.

**Methods:**

A descriptive and observational case-control study was carried out in universities that offer nursing designation. A total sample of 140 freshman nursing students was recruited. Concretely, 70 participants were recruited from an university receiving hybrid education and 70 participants were recruited from another university receiving virtual learning education. Nursing students self-reported the following questionnaires: the Rosenberg Self-Esteem scale, the 10 CD-Risk Connor-Davidson Risk Resilience Scale, the Beck Anxiety Inventory, the Beck Depression Inventory and the Academic Stress Coping Scale.

**Results:**

Significant differences (P = 0.001) showed higher depression levels for students who received virtual education versus received hybrid education. Differences in Rosenberg test for self-esteem and Beck Anxiety Inventory for anxiety (P > 0.05) were not found.

**Conclusion:**

Students who received online education presented higher depression levels which could be due to they had not face-to-face relationships with teachers or classmates, whereas the hybrid education group received a different type of direct interaction with teachers and peers.

## Background

Worldwide, the coronavirus 2019 (COVID-19) pandemic led to deep social changes and especially nurses suffered from mental health disorders [1]. Considering the university environment, especially in nursing students, the first episode of COVID-19 supposed a teaching revolution due to affected students received mainly online education [2, 3]. The initial online model, which provides isolated online education without face-to-face classes, has given rise to a hybrid model [4], which comprised education including the half of the students receiving face-to-face classes and another half of students receiving online classes at the same time, rotating weekly. Nevertheless, not all universities have dared to implement the hybrid education model due to fear of infection, leaving only face-to-face classes for internships and clinics. Regarding the university environment, students feel abandonment, sadness, lack of motivation, and academic performance below the average with respect to the academic results prior to the pandemic [5, 6].

Concretely, nursing students’ quality of life was negatively influenced by academic stress and anxiety during COVID-19 pandemic, although resilience may mediate this impact to preserve their quality of life [7]. Indeed, resilience may be considered as a mediator to minimize the academic stress effects on self-directed learning [8]. Greater anxiety and depression levels were linked to being a first-year nursing student [9]. Furthermore, nursing students who experienced low self-esteem presented higher association with depressive symptoms [10].

Currently, there is an urgent need to develop emergency and preventive measures to address the mental health of university students [11] since students have begun to show alarming rates of depression, anxiety and/or suicidal thoughts in addition to academic, health, and lifestyle alterations caused directly by the pandemic [12]. Regarding the universities of Madrid (Spain) which include first-year nursing students, some studies have focused on psychological factors secondary to the educational restrictions of COVID-19 [13–15]. Thus, this study was conducted due to online learning received by freshman students during the COVID-19 pandemic may promote academic stress [7], anxiety and depression [9], associated to low self-esteem [10] and resilience [8], while a hybrid learning comprised by a mixed model of combined face-to-face and online classes could minimize this psychological and academic impact [4]. Nevertheless, there is a lack of studies comparing nursing university students who received isolated online education with respect to nursing students from another university who received hybrid education alternating face-to-face lectures with online classes weekly throughout the same academic year. Therefore, it is for these reasons that the main objective of this research was to establish whether a relationship exists between both groups considering the following factors: levels of resilience, self-esteem, anxiety, depression, and academic stress. Thus, it is hypothesized that students who receive all lectures online will have higher levels of unseeded depression than the group who attend face-to-face lectures every other week on campus.

## Materials and methods

### Study Design

A descriptive and observational case-control study was carried out in 2 different Spanish universities called the Universidad Rey Juan Carlos (URJC) and the Universidad Complutense de Madrid (UCM) in Madrid-Spain, Schools of Nursing Degree in October 2021. The Strengthening the Reporting of Observational Studies in Epidemiology (STROBE) checklist and criteria for case-control studies were followed [16].

### Setting and sample

Calculation for sample size determination was carried out considering the difference for 2 independent groups utilizing the G*Power software (version 3.1.9.2). In addition, a two-tailed hypothesis, an effect size of 0.50 and an α error probability of 0.05 with a β level of 20% were used. Lastly, a desired power analysis of 80% (1-β error probability) and an allocation ratio (N2/N1) of 1 were used for the sample size calculations. Therefore, a total sample size of 128 participants was calculated with at least 64 participants per group. The sample was recruited through a consecutive sampling method using a successive simple method by the same online survey for both URJC and UCM universities, obtaining up to 140 freshman nursing degree university students. Subjects were enlisted from freshman students from URJC receiving hybrid education and students from UCM receiving virtual education. Both groups were matched according to age (19 years old) and sex (all participants were female nursing freshman university students). Participation selection and inclusion criteria included several parameters: (1) female students of 19 years old; (2) subjects enrolled in the freshman nursing degree class at URJC and UCM, and (3) single subjects without children or dependents who were living with their families. Exclusion criteria comprised male and non-freshman nursing students, aged different from 19 years old, who studied in other universities different from UCM and URJC, native speakers different from Spanish language, and non-single participants with children or dependents.

### Ethical consideration

The ethics committee of Universidad Rey Juan Carlos (code: 2,910,202,121,221) approved this research, and all subjects signed the informed consent form before the beginning of the study. Furthermore, the declaration of Helsinki was considered and rules for human experimentation were taken into account.

### Procedure

Data collection was carried out using the same online survey which was completed by both groups for approximately 45–60 min in October 2021. This online method for both groups overcame common biases of face-to-face surveys. The feasibility, applicability, and clarity of the research tools were previously stated due to all questionnaires were valid, reliable and transculturally adapted to Spanish language. Permissions to use these tools were not required due to these questionnaires were available without any fee or copyright. Thus, a pilot study was not necessary because the clarity of all items were reported in previous studies. Baseline measurements were self-reported including general questions associated with demographic variables: (1) sex (all students were female), (2) weight, (3) height, (4) body mass index ([BMI] kg/cm^2^) and (5) all students aged 19 years old.

Freshman students from both universities took the same lectures on campus during the first and second semesters and same weeks of pre-clinical rotation in the second semester according to education schedule requirements of Madrid government, Spain. One group received all lectures as virtual mode using a team platform called “Aula Virtual URJC” [17] and another one giving lectures by alternating one week using a face-to-face format on campus and the following week using a virtual format with the platform called “Hybrid UCM” [18] to compare them. Both universities presented similar nursing schools due to they were public institutions from the same rigorous criteria for evaluation according to the Madrid regional government and Spanish National Agency for Quality Assessment and Accreditation. In addition, students from both public universities presented a similar medium socio-economic status due to both nursing schools required similar financial fees and access requirements.

Next, participants completed the following test the same day at both universities at the end of academic year (freshman). Freshman students were new to the university experience. They were students who arrived from high school and who had also experienced virtual teaching and confinement during the acute phase of the pandemic.

### Measurements

*Rosenberg Self-Esteem Scale (RSE)* [19, 20]. The RSE questionnaire consists of 10 questions, scored from 1 to 4 points, (4 = strongly agree, 3 = agree, 2 = disagree, 1 = strongly disagree). Indeed, 5 statements present positive direction, as well as other 5 statements show negative direction. The authors of the questionnaire set limits for this scale, but a range of scores between 20 and 30 points is usually considered the normal range. If the score was greater than the normal range, such as result would indicate high self-esteem, whereas if the result was less than normal, low self-esteem is indicated. The scale has high reliability with test–retest correlations in the range of 0.82 to 0.88 [19] and 0.87 for Spanish population [20].

*Ten CD-Risk, Connor-Davidson Risk Resilience Scale (CD-RISC)* [21, 22]: Resilience was evaluated using the short version of 10-items CD-RISC that was validated in Spanish by Notario-Pacheco et al. [21]. The scale consists of 10 items which corresponded to those numbered 1, 4, 6, 7, 8, 11, 14, 16, 17 and 19 from the original scale designed by Connor and Davidson [22]. The other numbered items were removed in this short version. Participants self-reported the most suitable response for each question of the Spanish validated scale [21]. The response format is a five-point Likert-type scale from 0 (totally disagree) to 4 (totally agree).The final score is the sum of all the responses obtained for each item (range from 0 to 40 points), and greater scores show higher resilience level. The reliability of 10-items CD-RISC is defined by a Cronbach’s α of 0.85, and the weights in factor analysis are within the range of 0.48 to 0.76.

*Beck Anxiety Inventory (BAI)* [23, 24]. The BAI questionnaire contains a list of 21 symptoms indicating anxiety with a 4-point Likert scale ranging from not at all to severe, and the degree to which each symptom affected them during the last week. Scores for each element were added up, as well as the total score ranged 0–63 points. If total scores ranged 0–7, a minimum anxiety level was considered; if total scores ranged 8–15, mild anxiety level was obtained; if total scores ranged 16–25, moderate anxiety level was obtained, and finally if total scores ranged 26–63, severe anxiety level was established [23]. Also, in the version adapted for the Spanish population, the instrument showed a high internal consistency with a Cronbach’s α coefficient of 0.92 and a test–retest reliability of 0.75. The BAI has a high internal consistency (Cronbach’s α from 0.90 to 0.94). The correlation between the items and the total score ranged from 0.30 to 0.71. The test–retest reliability after one week ranged from 0.67 to 0.93, and after seven weeks, the reliability was 0.62 [24].

*Beck Depression Inventory (BDI, BDI-II)* [25, 26]. The BDI is questionnaire with a group of 21 items, and all questions use a Likert scale for answers. Indeed, internal consistency showed an α of 0.78. Items of the sample (i.e. sadness) presented responses such as “I don’t feel sad” or “I feel sad most of the time”. Indeed, the original version of the BDI-II manual [25] considered cut-off values and depression grades such as: (1) minimal depression ranged from 0 to 13 points, (2) mild depression ranged from 14 to 19 points, (3) moderate depression ranged from 20 to 28 points, and (4) severe depression ranged from 29 to 63 points. The Spanish adaptation of Sanz and Vázquez [26] assumes the cut-off scores designed by Beck et al. [25], and the reliability of the instrument is high both in terms of internal consistency (Cronbach’s α coefficient = 0.83) and temporal stability (test–retest correlations ranged between 0.60 and 0.72 for 3 different subgroups regarding the total sample).

*Academic Stress Coping Scale (ASCS)* [27]. The ASCS scale is a subscale of the Academic Stress Questionnaire (CEA) questionnaire, an instrument made up of three scales, which is used to assess academic stressors, stress responses, and stress coping strategies. The ASCS scale is made up of 23 items, formulated to evaluate the cognitive and behavioral strategies used by the student when facing situations of academic stress. It is a scale with Likert-type responses to each item for which the student can choose between five options: Never (1), Sometimes (2), Quite a few times (3), Many times (4), and Always (5). The reliability of the ASCS scale has a general Cronbach’s coefficient for this study of 0.885. In turn, this coping scale is divided into three factors that are specified below: (1) Factor 1 (1 COP). Positive Reassessment: This dimension groups nine items (1, 3, 4, 5, 6, 14, 17, 18, and 19) that present different ways of coping aimed at creating a new positive meaning about the problem or academic difficulty. This factor underlines its active and positive character in propositions of direct quotes such as “When I face a problematic situation the night before the exam, I try to think that I am prepared to do it well” or “When I face a complicated situation, in general, I try not to give it importance to the problems” [27]. Its internal consistency according to Cronbach’s α was 0.668; (2) Factor 2 (COP 2). Search for Social Support includes seven items (2, 8, 10, 13, 20, 21, and 23) to assess active and behavioural coping based on the student’s search for information and advice as social support for the problem and also for understanding by other people as emotional support for what they are experiencing. Its internal consistency according to Cronbach’s α was 0.727; and (3) Factor 3 (COP 3). Planning and management of personal resources includes seven items (7, 9, 11, 12, 15, 16, and 22) that refer to the activation of strategies based on analysis and reason to change the problematic situation and that denotes a type of behavioural and active coping. Its internal consistency according to Cronbach’s α was 0.741 for this study.

### Data Analysis

Regarding quantitative data, all variables were examined for normality of distribution using the Kolmogorov–Smirnov test, and data were considered normally distributed if P > 0.05. Descriptive analyses, including calculation of means, standard deviations (SD), and 95% confidence intervals (CI) were calculated for quantitative variables according to normal distributions. Median and 95% CIs were described for non-normally distributed data. For categorical data, frequencies as well as % were used to describe these values. Differences between groups were contrasted using independent Student’s t considering the Levene´s test for equality of variances or Mann–Whitney U tests when variables showed normal or non-normal distribution. Differences between groups were compare using the chi-square test for qualitative variables. In addition, linear regression analyses were performed to predict the Beck Depression Inventory (BDI, BDI-II) levels as the outcome measurement that showed statistically significant differences between both groups. These linear regression analyses were performed using the *R*^2^ coefficient to quantify the adjustment quality according to the pre-established values for F probability (*P*_in_ = 0.05, *P*_out_ = 0.10). Descriptive data which showed statistical differences between both groups (height and weight) were considered independent variables. Depression levels (BDI, BDI-II) were considered the dependent variable. In all analyses, statistical significance was established with a *P*-value < 0.05 with a 95% CI. Lastly, each analysis was carried out with the statistical software SPSS (using the version 19.0; from Chicago, IL, USA).

## Results

A total of 140 female participants aged 19 years old were recruited in the study with 70 participants in hybrid learning education and 70 participants in the virtual group. Female participants of the total sample showed a median of weight of 60 kg, height of 165 cm and BMI of 22.30 Kg/m^2^. Indeed, the variables of height, weight, and BMI showed a non-normal distribution (P < 0.05) in both groups demonstrating statistical differences between groups for height and weight (P < 0.05), but not for BMI (P = 0.094). These data were shown in Table 1.

**Table 1:** Descriptive data of the participants in hybrid and virtual education matched-paired by age (all participants were 19 years old and female nursing freshman university students)

Descriptive Data	Total Group N = 140		HIBRID UCM Mean ± SD (95%CI) n = 70		VIRTUAL URJC Mean ± SD (95%CI) n = 70		*p*-Value
	Mean ± SD (95%CI)	Median (95%CI)	Mean ± SD (95%CI)	Median (95%CI)	Mean ± SD (95%CI)	Median (95%CI)	
Weight (kg)	61.08 ± 11.52 (59.15–63.01)	60.00 (59.00–65.00)	58.47 ± 10.29 (56.01–60.92)	56.50 (54.00–60.00)	63.73 ± 12.15 (60.81–66.85)	65.00 (60.00–70.00)	0.005*
Height (cm)	164.52 ± 15.80 (161.87–167.17)	165.00 (165.00-168.00)	163.68 ± 7.20 (161.96–165.40)	164.50 (160.00-165.00)	165.37 ± 21.28 (160.26–170.48)	168.50 (165.00–173.45)	0.005*
BMI (Kg/m^2^)	22.05 ± 3.13 (21.53–22.58)	22.30 (21.50–22.90)	21.68 ± 3.10 (20.94–22.93)	21.50 (20.30–22.97)	22.43 ± 3.14 (21.68–23.19)	22.50 (21.90–23.08)	0.094*

Abbreviations: BMI, body mass index; Kg, kilograms; Cm, centimetres; SD, standard deviation; CI, confidence interval and * U Mann Whitney test for independent groups were applied; p < 0.05 with a 95% confidence interval was considered statistically significant

Quantitative scores of the ASCS and 10 CD-RISC were described as mean, median and 95% CI in hybrid and virtual education and did not show differences between groups (P > 0.05) in resilience and academic stress domains such as positive reassessment, search for social support planning and management of personal resources. These results concerning the ASCS and 10 CD-RISC were presented in Table 2.

**Table 2:** Confidence interval (95%), mean, median and interquartile range scores in cases and controls for ASCS and 10 CD-RISC questionnaires

Groups	Hybrid UCM (n = 70)		Virtual URJC (n = 70)		
Item	Mean ± SD (95%CI)	Median (95%CI )	Mean ± SD (95%CI)	Median (95%CI )	*p*-Value *
10 CD RISC: Resilience	26.31 ± 3.82 (25.39–27.23)	26.00 (26.00–28)	26.05 ± 4.24 (25.04–27.07)	27.00 (25.00–29.00)	0.796*
ASCS COP 1: Positive Reassessment	2.90 ± 0.59 (2.76–3.05)	2.77 (2.66–3.11)	2.89 ± 0.71 (2.71–3.06)	2.77 (2.59–3.00)	0.874*
ASCS COP 2: Search for Social Support	2.93 ± 0.85 (2.73–3.14)	2.83 (2.50–3.33)	2.89 ± 0.74 (2.72–3.07)	2.83 (2.72–3.00)	0.865*
ASCS COP 3: Planning and management of personal resources	3.04 ± 0.76 (2.86–3.23)	3.14 (2.82–3.42)	2.95 ± 0.70 (2.78–3.12)	3.00 (2.76–3.14)	0.277**

Abbreviations and interpretation: SD, standard deviation; CI, confidence interval; 10 CD-RISC; Ten CD-Risk, Connor-Davidson Risk Resilience Scale, highest scores indicate the highest level of resilience, Academic Stress Coping Scale: ASCS COP 1: Positive Reassessment, highest scores indicate the highest level of resilience, ASCS COP 2: Search for Social Support, highest scores indicate the highest level of resilience; ASCS COP 3: Planning and management of personal resources, highest scores indicate the highest level of resilience; *P value from U Mann Whitney and ** Independent t Student test were applied for independent groups. In all analyses, p < 0.05 with a 95% confidence interval was considered statistically significant

Categorical test scores from the hybrid and virtual education groups showed significant differences in levels of depression, which were higher in the virtual URJC group (P = 0.001). Nevertheless, there were not differences in Rosenberg test of self-esteem and BAI for anxiety (P > 0.05). These findings were shown in Table 3.

**Table 3:** Comparisons of categorical test scores between hybrid and virtual educations groups

Outcome measurements		Hybrid UCM (n = 70)	Virtual URJC (n = 70)	*p*-Value
Test Rosenberg	Low (< 20) Normal (20–30) High (> 30)	19(27.1%) 51(72.8%) 0(0.0%)	13(18.5%) 57(81.4%) 0(0.0%)	0.314
Beck Anxiety Inventory (BAI) Anxiety	Minimum (0–7) Mild (8–15) Moderate (16–25) Severe (26–63)	20(28.5%) 13(18.5%) 24(34.2%) 13(18.5%)	17(24.2%) 22(31.4%) 13(18.5%) 18(25.7%)	0.084
Beck Depression Inventory (BDI, BDI-II). Depression	Minimum (0–13) Mild (14–19) Moderate (20–28) Severe (29–63)	33(47.1%) 24(34.2%) 11(15.7%) 2(2.8%)	44(62.8%) 5(7.1%) 16(22.8%) 5(7.1%)	**0.001**

Abbreviations and interpretation: Test Rosenberg, Lower than 20 = Low self-esteem; between 20 and 30 = normal; Higher than 20 = high self-esteem. Beck Anxiety Inventory (BAI): Lower or equal to 7 = Minimum level of anxiety; Mild = 8–15; Moderate = 16–25; Severe = equal or higher than 26; Beck Depression Inventory (BDI, BDI-II). Minimum level of depression = 0–7: Mild = 8–15; Moderate = 20–28; severe = 29–63; Frequency, percentage (%) and chi-squared test (χ^2^) were utilized. In all the analyses, p < 0.05 (with a 95% confidence interval) was considered statistically significant (bold)

Finally, linear regression models showed that levels of depression (BDI, BDI-II) were not predicted nor influenced by weight (*R*^2^ = 0.004; β = 0.005; F_1,138_ = 0.561; P = 0.455) nor height (*R*^2^ = 0.018; β = -0.008; F_1,138_ = 2.589; P = 0.110), which indicated that demographic differences in our sample did not influence depression findings in our study.

## Discussion

In our study, the objective was to analyse the levels of resilience, academic stress including their domains of positive reassessment, search for social support, planning and management of personal resources, as well as self-esteem, anxiety, and depression in freshman students, who were new to the university experience, from two universities offering different education formats, one group received all lectures in the virtual mode and the other one presented an alternating form of lectures with one week face-to-face on campus and the following week virtual to compare them. Our hypothesis stated that students receiving online lectures would have worst results that the group receiving face-to-face lectures on campus every other week.

The results showed that there were some differences between the two groups in terms of weight and BMI since the online group showed higher scores for both variables. Studies have previously shown that the lack of physical activity and sedentary lifestyle caused by an online education, which avoided daily socialization secondary to university attending, could be the cause of weight gain and higher BMI [28, 29]. These previous studies with adolescents have shown that lifestyle changes during the COVID-19 lockdown (mandatory online education, school closure, increased sleep time, and more accessible cyberspace) led to worsening in health-related behaviours, such as physical activity and diet [28, 29].

Considering differences between groups with respect to self-esteem, resilience, anxiety, depression, and academic stress coping domains, our findings suggested significant differences in higher depression levels for online learning compared to hybrid learning modalities. These results supported our hypothesis that depression scores between two groups could present differences with higher scores for students who received online education compared with the students who received hybrid education. In line with our study findings, a prior study carried out in university students from Bangladesh reported that psychological alterations, including depression as a main focus, affected university students secondary to social restrictions of isolated online education [30]. According to Chen and Lucock [31], the COVID-19 pandemic impaired mental health status of university students showing high levels of anxiety and depression by an online survey completed by 1173 students at one university from the North of England. Thus, university students suffered from psychological alterations, such as depression, which may be influenced by several conditions, including family stress, family and peer pressure, lack of financial support, high parental expectations, lack of sleep, inappropriate internet use, increased screen time, isolation, toxic psychological environment, academic pressure, as well as demanding workloads and exam schedules [32].

With respect to the rest of outcome measurements, online and hybrid education modalities did not present differences for resilience and academic stress domains scores, such as positive reassessment, search for social support planning and management of personal resources, as well as self-esteem and anxiety levels. Although online education in freshman nursing students during the COVID-19 pandemic may increase academic stress and anxiety, mediated by low resilience [7] and self-esteem [10], our study findings suggested that adding face-to-face to online classes by a mixed hybrid model did not minimize this psychological and academic impact [4]. Nevertheless, these results may be also due to the use of different tools such as specific COVID-19 Anxiety and Perception of Academic Stress scales [7], as well as nursing students from different countries cultures [10].

### Implication

Our study findings presented key implications to improve the mental health of nursing university students suffering from higher depression levels according to prior studies [33, 34]. We propose the use the levels of self-reported depression questionnaires in freshman nursing students in order to promote hybrid education modalities versus isolated online learning methods to alleviate depression symptoms [4]. Specially, these psychological alterations were increased during the COVID-19 pandemic and hybrid learning strategies should be considered to improve quality and academic performance in future pandemic environments [30, 31].

### Limitation

Nevertheless, this research presented some limitations. The following factors were not under control which may affected our study findings, such as sleep habits, physical activity, and diet. In addition, the aforementioned co-variables such as family stress, peer and academic pressure, poor financial support, parental expectations, lack of sleep, excessive internet use, isolation, psychological environment and academic pressure and schedule, could influence our results and should be taking into account for future studies [32]. Finally, external validity of our study findings cannot be extra-poled to other cultures, being necessary to replicate this study in other counties [10].

### Strengths

The sample size and sampling methods were adequate and designed in accordance with a descriptive and observational case-control study design following the STROBE checklist and criteria for case-control studies in an accurate manner [16]. In addition, the internal validity of our study was reinforced by the use of Spanish validated and reliable tools which were previously tested in this population, including RSE [19, 20], 10 CD-RISC [21, 22], BAI [23, 24], BDI-II [25, 26] and ASCS [27] questionnaires.

## Conclusions

In conclusion, freshman nursing university students who received isolated online education during COVID-19 pandemic presented higher levels of depression, which could be due to they had not face-to-face relationships with teachers or classmates, whereas the hybrid education group received a different type of direct interaction with teachers and peers. Nevertheless, both online and hybrid education modalities did not present differences regarding resilience and academic stress domains scores, such as positive reassessment, search for social support planning and management of personal resources, as well as self-esteem and anxiety levels of nursing students.

Therefore, we recommend hybrid education modalities versus isolated online learning methods to alleviate depression levels in freshman nursing students, especially in pandemic environments.

## Data Availability

The datasets used and/or analysed during the current study are available from the corresponding author on reasonable request.

## Abbreviations

ASCS: Academic Stress Coping Scale
BAI: Beck Anxiety Inventory
BDI, BDI-II: Beck Depression Inventory
BMI: Body mass index
CD-RISC: Connor-Davidson Risk Resilience Scale
URJC: Universidad Rey Juan Carlos
UCM: Universidad Complutense de Madrid
RSE: Rosenberg Self-Esteem Scale Ten CD-Risk
